# More than two years delay in the union of fracture neck of femur after primary intervention

**DOI:** 10.1186/1757-1626-1-61

**Published:** 2008-07-25

**Authors:** Naseem Ul Gani, Khursheed Ahmed Kangoo, Mohammed Farooq Butt, Gulam Nabi Dar, Mudassir Maqbool Wani

**Affiliations:** 1 Department of orthopedics, Govt hospital for bone and joint surgery Barzullah, Srinager, India; 2 Department of surgery, GMC, Srinagar, India

## Abstract

**Introduction:**

More than two years delay in the union of fracture neck of femur is a very rare entity.

The treatment of an established non union depends on numerous factors including age of the patient, vascularity of the femoral head and other factors. It is timing of intervention that is not clearly defined in the literature.

**Case presentation:**

We report 2 cases where fracture neck of femur in 2 Asian males of 37 and 52 years of age took more than 2 years to unite after primary intervention.

**Conclusion:**

We believe if the implant is holding and patient is able to bear some weight, some of these fractures may unite without any further intervention.

## Background

The standard treatment for displaced fracture of neck of femur in young adults is internal fixation, aimed at salvaging the femoral head [1]. Nonunion is a frequent complication of intracapsular fractures of the femoral neck because healing of a fracture neck of femur is different from other long bones. Blood supply is precarious which is further jeopardized by displacement of fragments and tamponade effect adds to the insult by occluding the veins at lower pressure and arteries at higher pressure. Because of the elongated position of the femoral neck within capsule and absence of cambium layer of periostium [2], the fracture neck of femur heals without external callous. Being intracapsular it is bathed by synovial fluid. Common to all intracapsular fractures, the synovial fluid bathing the fracture may interfere with healing process. Angiogenic inhibiting factors can also inhibit fracture repair [3]. In combination all these factors lead to complications like nonunion, avascular necrosis and segmental collapse of the femoral head [4]. The stability of the fixation depends on the accuracy of reduction, the implant used and density of the bone [5]. Non union may occur where fixation stability has been compromised by poor surgical technique [6]. Nonunion can be diagnosed within 12 months of fracture fixation [5]. After nonunion has been established, intervention is inevitable. We report 2 cases of nonunion fracture neck of femur who refused further intervention at one year after fracture fixation and were allowed to continue with partial weight bearing as the implant was holding. Both patients consented to a regular follow up and were supervised carefully. Both fractures ultimately united after 2 years.

## Case reports

### Case no 1

A 38 year old Kashmiri male businessman with displaced fracture neck of femur was admitted in our hospital. He was operated within 48 hours. A good reduction and fixation was achieved using 3 AO 6.5 mm cannulated screws. Patient was allowed partial weight bearing as per pain tolerance in post operative period. At 6 months patient complained of a persistent groin pain on ambulation. Radiographs at 6 months showed nonunion. Patient was supervised till one year with the hope that the fracture might unite. But radiographs at one year showed clear cut gap at fracture site and there was no progress towards fracture healing. However there was no malalignment or shortening. Implant was holding and had not displaced. Head sphericity was maintained and there was no loss of articular cartilage space. At this time surgical options were discussed with the patient. As patient had mild pain, he refused surgery and preferred to use a stick and consented to 2 monthly follow up. After 26 months of fracture fixation patient's pain subsided and radiograph at 32 months showed full union. The patient at this moment was assymptomatic, had good range of motion around hip and knee and had no shortening.

### Case no 2

**Figure 1:**
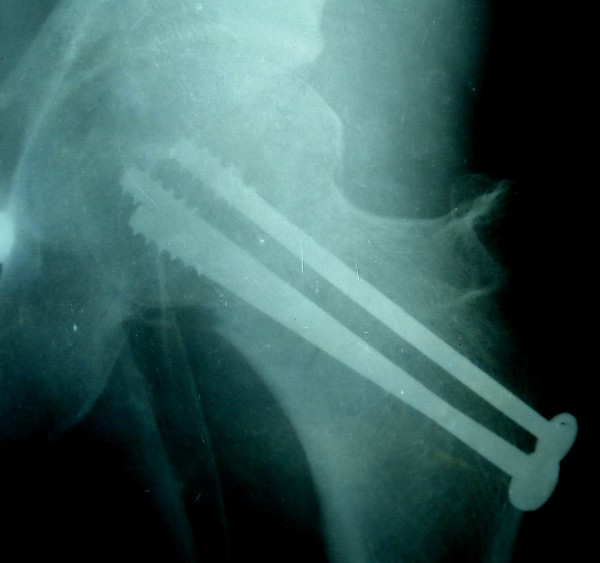
Case no 2, immediate Post operative radiograph AP view.

**Figure 2:**
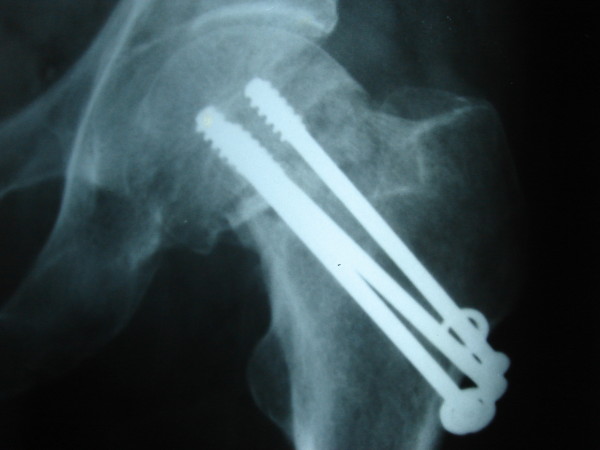
Case no 2, radiograph at 6 months, showing displacement.

**Figure 3:**
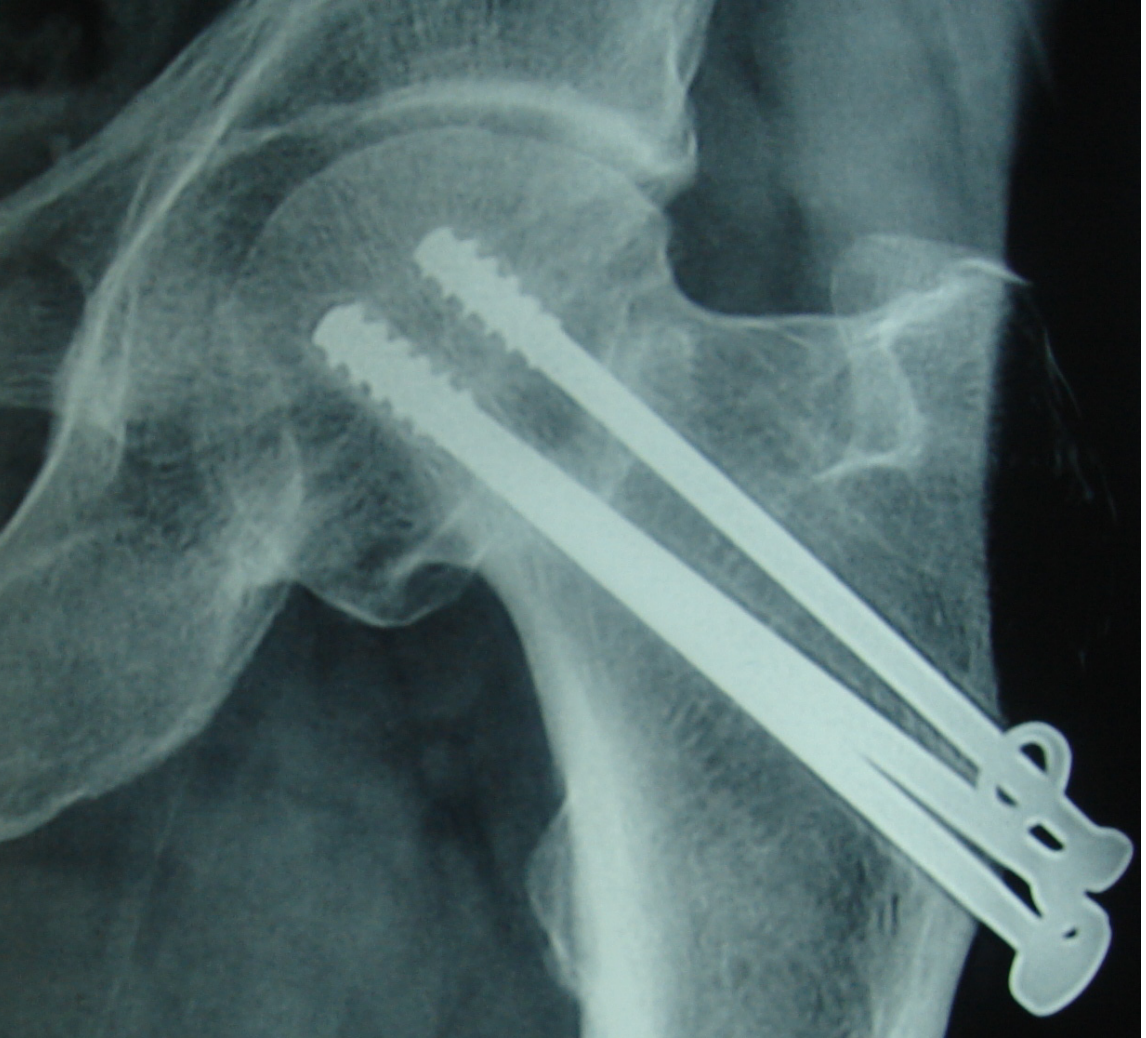
Case no 2, radiograph showing union at 29 months.

A 52 year old Kashmiri male farmer from a far flung area of our state reported to our hospital 6 days after sustaining fracture neck of left femur. Patient was operated within 2 days. Closed reduction and internal fixation was done using three cannulated screw (Fig. 1). In this patient as a result of surgical error, screws were not parallel in lateral view. After discharge from the hospital, patient was lost in follow up for 6 months. At 6 months patient had pain in the affected hip on weight bearing. Radiographs showed that the fracture had displaced from its original position and had varus angulation (Fig. 2). Since the implant was holding patient was advised touch down weight bearing. Patient was discharged and asked for a regular follow up every 2 months. At one year patient was bearing partial weight on the limb and was using a stick. Clinically patient had pain in the affected hip on weight bearing. Radiographs showed no further displacement of the fracture though there was a gap at the fracture site. Patient refused any surgical intervention at this time and agreed to a regular follow up. Over a period of one year patients symptoms improved and radiographs after 29 months of fracture fixation showed union (Fig. 3). Patient had no pain on weight bearing and had a good range of motion of affected hip and ipsilateral knee though he had shortening of 1.5 cm.

## Discussion

The standard treatment for displaced fracture of the femoral neck in young adults is internal fixation, aimed at salvaging the femoral head [1]. Rate of non union in fracture neck of femur has been reported to be 2 to 22% and generally becomes apparent within one year [2,7-9]. Delayed union or nonunion often manifests as continued pain localized to the groin and over anterolateral aspect of injured hip aggravated by weight bearing. The treatment of an established nonunion depends on numerous factors including the age of the patient, vascularity of the femoral head; alignment of the neck and shaft and potential limb length discrepancy [5]. However timing of such an intervention is not clearly defined in the literature. In elderly symptomatic patients preferred treatment is endoprosthetic replacement. In the young adults, femoral head salvage is almost always indicated [1]. There are numerous surgical procedures to achieve union with preservation of the femoral head. Replacement of cancellous screws with a larger screw may be considered in the absence of malalignment, shortening, loss of reduction and avascular necrosis [5]. This could have been the treatment of case no 1 at one year. Since patient refused surgery at one year, we had no option but to continue with supervised neglect of the patient. This case demonstrated as long as the implant is holding, good results may be achieved even after two years of fracture fixation.

Fixation combined with proximal femoral osteotomy is indicated in the treatment of nonunion with shortening or varus angulation [5]. This could have been the treatment of case no 2. Again patient refused surgery, so we had to continue with conservative treatment. This case demonstrated that fracture neck of femur which had displaced after fixation can still unite though it can delay up to 2 years.

## Conclusion

We conclude that as long as implant is holding, patient is able to bear some weight and patient is ready for a regular follow up, union can be achieved even after 2 years of fixation of a fracture neck of femur without any further intervention.

## Competing interests

The authors declare that they have no competing interests.

## Authors' contributions

NUG designed the study, wrote the manuscript, performed literature review, KAK, MFB, GND, MMW helped in literature review and drafting the final manuscript. All authors read and approved the final manuscript.

## Consent

Written informed consent was obtained from the patients for publication of this case report and accompanying images. A copy of the written consent is available for review by the Editor-in-Chief of this journal.
